# *Journal of Eye Movement Research*: Opening a New Chapter with MDPI

**DOI:** 10.3390/jemr18010001

**Published:** 2025-01-16

**Authors:** Rudolf Groner

**Affiliations:** Department of Psychology, University of Bern, 3012 Bern, Switzerland; rudolf.groner@unibe.ch

In 1980, together with Dieter Heller, I initiated an interdisciplinary network called the “European Group of Scientists active in Eye Movement Research”, including scientists who use eye movement registration as a research tool, developing models based on oculomotor data obtained from a wide spectrum of phenomena, ranging from the neurophysiological to the perceptual and the cognitive levels. The group’s focus was on exchanging information on the current research, equipment, and software. Starting in 1981, it organized biennial conferences (ECEM, https://www.eyemovement.org/, accessed on 2 January 2025) at different locations all over Europe. The group published edited books and, starting in 2007, launched the *Journal of Eye Movement Research* (*JEMR*), an interdisciplinary, peer-reviewed, scientific online journal.

After 17 years of cooperation with BOP (Bern Open Publishing) it was noted that, in order to stay successful, it seems necessary for a journal to coordinate with potential competitors within a strong publishing company. After a careful selection process, it was decided in June 2024 to choose MDPI as the new publisher of *JEMR* [1].

I am proud to have served the chief editor of the journal from its very beginning. Throughout this journey, I have been supported in my work by Simon Raess, Eva Krueger-Siegenthaler, and Walter Bischof, Sarah Singer, and Peter Stoffel. However, probably the most important input in terms of methodological and substantial questions came from my wife, Marina Groner. Due to my age and state of health, I will now resign from my post as soon as a successor has been elected.

My best wishes to the journal under its new publisher and to the Editorial Board, for continued success.
